# Quantitative Evaluation by Glucose Diffusion of Microleakage in Aged Calcium Silicate-Based Open-Sandwich Restorations

**DOI:** 10.1155/2012/105863

**Published:** 2011-12-12

**Authors:** S. Koubi, H. Elmerini, G. Koubi, H. Tassery, J. Camps

**Affiliations:** ^1^Department of Operative Dentistry, Faculty of Dentistry, Université de la Méditerrannée, 25 Boulevard Jean Moulin, 13005 Marseille, France; ^2^Department of Operative Dentistry, Faculté d'Odontologie, Université de la Méditerranée, 25 Boulevard Jean Moulin, 13005 Marseille, France; ^3^Department of Operative Dentistry, Faculty of Dentistry, Casablanca, Morocco; ^4^Department of Biomaterials, Faculté d'Odontologie, Université de la Méditerranée, Marseille, France

## Abstract

This study compared the *in vitro* marginal integrity of open-sandwich restorations based on aged calcium silicate cement versus resin-modified glass ionomer cement. Class II cavities were prepared on 30 extracted human third molars. These teeth were randomly assigned to two groups (*n* = 10) to compare a new hydraulic calcium silicate cement designed for restorative dentistry (Biodentine, Septodont, Saint Maur des Fossés, France) with a resin-modified glass ionomer cement (Ionolux, Voco, Cuxhaven, Germany) in open-sandwich restorations covered with a light-cured composite. Positive (*n* = 5) and negative (*n* = 5) controls were included. The teeth simultaneously underwent thermocycling and mechanocycling using a fatigue cycling machine (1,440 cycles, 5–55°C; 86,400 cycles, 50 N/cm^2^). The specimens were then stored in phosphate-buffered saline to simulate aging. After 1 year, the teeth were submitted to glucose diffusion, and the resulting data were analyzed with a nonparametric Mann-Whitney test. The Biodentine group and the Ionolux group presented glucose concentrations of 0.074 ± 0.035 g/L and 0.080 ± 0.032 g/L, respectively. No statistically significant differences were detected between the two groups. Therefore, the calcium silicate-based material performs as well as the resin-modified glass ionomer cement in open-sandwich restorations.

## 1. Introduction

Calcium silicate cements, such as the Portland cement, possess good mechanical properties but are not commonly used in medicine because their long setting times render them unsuitable for clinical applications; maximum compressive strength is achieved after 28 days [1]. In addition, they contain heavy metals [2], are not radiopaque [3], and have a large setting expansion [4]. Nevertheless, efforts have been made to overcome these problems, and several calcium silicate-based cements have recently been introduced in medicine and dentistry. In medicine, calcium silicates are mainly used as bone graft materials [5–7]. In dentistry, they have been tested for treatment of dentin hypersensitivity [8] and gave promising results in endodontics [9]. However, the most famous calcium silicate cement remains the mineral trioxide aggregate (MTA; Dentsply, Tulsa Dental Specialties, Johnson City, Ten, USA). MTA was introduced in 1993 [10] and is designed for root-perforation treatment, retrograde filling, and open-apex closure on immature teeth. Three excellent reviews have described the physical and bacteriological properties [11], the leakage and biocompatibility investigations [12], and the mechanisms of action [13] of MTA.

The Portland cements designed for medicine and dentistry, also called hydraulic silicate cements [14], mainly contain tricalcium silicate (3CaO-SiO_2_; C_3_S), because it is responsible for rapid setting and development of early strength and exhibits higher reactivity than the other calcium silicates [15]. Unfortunately the C_3_S-based materials that combine a fast setting time with outstanding biological properties have poor mechanical properties that make them inappropriate for restorative dentistry [16–18]. More recently, a fast-setting calcium silicate-based restorative material especially designed for restorative dentistry has been brought onto the market (Biodentine, Septodont, St Maure des Fossés, France). This material exhibits the same excellent biological properties as MTA and can be placed in direct contact with dental pulp [19], although its sensitivity to abrasion makes it a poor enamel substitute. However, Biodentine may be a good candidate for a dentin substitute in sandwich restorations: it does not require photoactivation and thus can be placed in bulk in the cavity, its setting time is short enough to complete the whole procedure in a single appointment, its mechanical properties are sufficient to withstand occlusal loading when protected with composite.

Leakage at the dentin material interface is responsible for postoperative sensitivity [20] and secondary caries [21]. Therefore, leakage of Biodentine which belongs to a new class of restorative material must be evaluated. It should preferably be compared with the resin-modified glass ionomer cements which have been utilized previously as a base material for this type of restoration in both laboratory and clinical investigations, with some degree of success [22]. To our knowledge, calcium silicate cements are not frequently used in restorative dentistry and their marginal integrity when used in open-sandwich restorations has never been tested. The purpose of this study was to compare the *in vitro* marginal integrity of open-sandwich restorations based on aged calcium-silicate cement versus resin-modified glass ionomer cement.

## 2. Materials and Methods

Thirty human third molars extracted due to dental crowding were used for this study. The teeth were collected and all remnants of soft tissue were immediately removed with gauze. The teeth were stored in 0.5% Chloramine-T at 4°C until usage within 2 months of collection. These experiments were performed according to national ethical laws. Only sound fully formed teeth lacking any sign of cracking due to the use of forceps were included in the study.

### 2.1. Specimen Preparation

After visual inspection with a light microscope to ensure that the teeth did not present caries or cracks due to extraction, the teeth were cleaned and polished with scalers and pumice. One standardized mesio-occlusal class II cavity was prepared on each tooth. All manipulations and restorations were performed by a single experienced operator to prevent variation due to operator skill. The operator performed these procedures under 3.5x magnification with fiber optic headlight illumination. Cavities were prepared with a high-speed handpiece, using a diamond bur (ISO 6856310023, Komet, Lemgo, Germany) under heavy water spray. The diamond bur was replaced after every five preparations. All internal line angles were rounded. The overall dimensions and depths of cavities were standardized as follows: occlusal floor, width 4 mm, length 5 mm; axial wall, width 4 mm, height 3 mm; gingival floor, width 4 mm, depth 2.5 mm. The proximal boxes ended in dentin, just below the enamel-cementum junction. The teeth were immediately and randomly divided into two groups according to the filling material used for the sandwich restoration: the Biodentine-group teeth (*n* = 10) were filled with calcium silicate-based Biodentine; the Ionolux-group teeth (*n* = 10) were filled with the resin-modified glass ionomer cement Ionolux. The negative control teeth (*n* = 5) were prepared and filled as in the Biodentine group but were entirely covered with two layers of nail varnish, and the positive control teeth (*n* = 5) were completely filled with TetricEvo Ceram (Ivoclar Vivadent, Schaan, Liechtenstein) without dentin wall treatment and without dentin-bonding agent between the dentin walls and the restorative material.

### 2.2. Cavity Filling

**Biodentine Group**

An Automatrix (Dentsply, Konstanz, Germany) was adjusted around the cavity and secured. The cavity was filled with Biodentine (Table 1) prepared according to the manufacturer's recommendations. After a 48 hours setting at 37°C and 100% humidity, a diamond bur mounted on a high-speed handpiece under copious water coolant was used to remove all material, leaving only the cervical third of the Biodentine filling on the gingival floor. The cavity was totally etched with 32% orthophosphoric acid for 15 s and thoroughly rinsed, and the enamel margins were etched for an additional 15 s and rinsed. The dentin walls were treated with All Bond 2 (Bisco, Schaumburg, Ill, USA) and the cavity was filled with three increments of TetricEvo Ceram composite. Each increment was light cured for 3 s, according to the soft polymerization concept, and finally cured for 40 sec. Composite was applied with a special instrument (CVHL1/2, Hu Friedy, Chicago, Ill, USA). The fiber optic headlight was turned off during the filling procedures to prevent premature partial polymerization of the light-curing material. The dentin-bonding agent and the composite were polymerized with a Blue phase 20i (Ivoclar-Vivadent) using a new 11-mm tip. The curing light was tested before each restoration and measured at least 1600 mW/cm^2^ each time on a curing radiometer (Demetron, Bioggio, Switzerland).

**Ionolux Group**

The smear layer covering the dentin walls was removed using 10% polyacrylic acid for 20 s. After rinsing for 20 s, an Automatrix was adjusted and secured. The resin-modified glass ionomer cement (Ionolux; Voco, Cuxhaven, Germany) was prepared according to the manufacturer's recommendations and placed in bulk to fill the cervical two-thirds of the cavity. The Ionolux was allowed to chemically set for 5 min and then was light-cured for 40 s; light curing did not occur immediately to permit material spreading and setting stress relaxation. The teeth were then prepared as described for the Biodentine group; cavity preparation leaving the cervical third filled with Ionolux, total etching, dentin bonding agent application, and cavity filling with TetricEvo Ceram.

### 2.3. Thermo-Mechanocycling

The teeth simultaneously underwent thermocycling and mechanocycling using a fatigue cycling machine (Proto-tech, Portland, Ore, USA) in conjunction with two recirculating water baths, a refrigerated bath (Merlin 33, Thermo Neslab, New Ington, NH, USA), and a heating bath (Isotemp 3016H, Fisher Scientific, Pittsburg, Pa, USA) [22]. A peristaltic water pump was used to return water from the teeth to the baths. These four devices were connected to three 3-way solenoid valves permitting adequate liquid circulation. Bath temperatures were self-regulated; the dwell time and the mechanical parameters were under control of the fatigue cycling machine. The dwell time was set at 30 s for 12 hours (720 complete cycles), and the bath temperatures were set at 5°C and 55°C. The teeth were mounted into acrylic potting rings, and the roots were partly embedded in epoxy resin (Buehler, Lake Bluff, Ill, USA) to secure the teeth. A guide rod, representing the stylus of the fatigue cycler, was used to adjust the specimen position so that the guide rod touched the restoration exactly where the round-ended stylus was placed during mechanocycling (in the center of the occlusal composite restoration). The acrylic rings containing the teeth were placed 5 by 5 in the mechanocycling device and secured for a 12-h thermo-mechanocyling. The loading device delivered an intermittent axial force of 50 N at 2 Hz for a total of 86,400 cycles.

### 2.4. Aging

Each group of teeth was stored for 1 year at 4°C in Dulbecco's phosphate-buffered saline with 0.5% chloramine-T added to prevent bacterial contamination (Table 2). The vials were frequently mixed and the liquid was renewed every 2 months. Aging was included to permit potential proximal dissolution of the dentin substitute.

### 2.5. Glucose Diffusion

**Figure 1:**
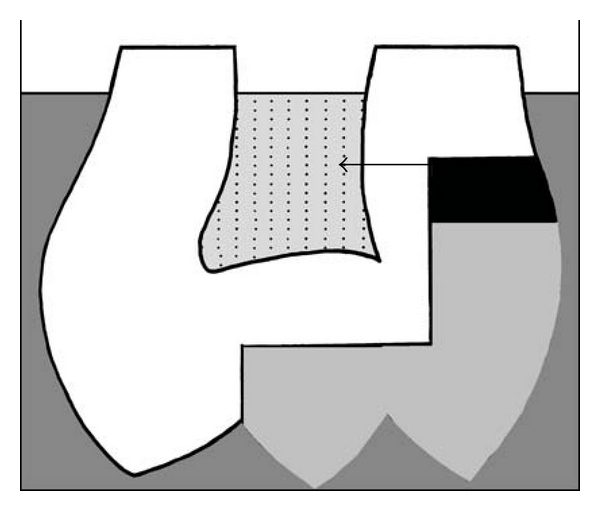
The glucose diffusion test. The tooth, with the lining material (in black) and the composite (in grey), was placed upside-down in 1 M glucose (dark grey), with the level of the solution below the tooth apex. Glucose diffused through the dentin-material interface and reached the pulpal space filled with water (dotted grey). The diffusion pathway is depicted by the arrow.

**Figure 2:**
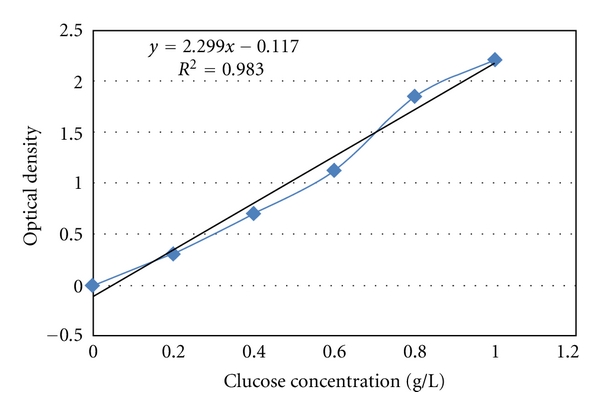
Standard curve of glucose concentration versus optical density at 540 nm.

The teeth were placed upside down in a plate and the pulp cavity was filled, through the open apices, with 60 *μ*L of bi-distilled water plus 0.2% NaN_3_. The plates were filled with 1 M glucose plus 0.2% NaN_3_ solution in water added such that he cavity margins were below the glucose solution level, but the apices were higher than the glucose solution level (Figure 1). The teeth were left in the plates for 1 h, allowing glucose diffusion from the glucose solution toward the pulpal space filled with bi-distilled water. Forty microliters of this liquid were collected from each tooth and placed in a 96-well plate. The preceding steps were all performed using high-magnification binoculars. The glucose concentration was determined with a glucose assay kit (Sigma Chemicals, St Louis, Mo, USA) according to the manufacturer's recommendations and recorded using a spectrophotometer at 540 nm. The results were expressed in g/L using a standard curve established prior to experimentation, from 0 to 100 *μ*g/mL (Figure 2). Since the sample size was less than 30, a nonparametric Mann-Whitney test was performed, at the 95% confidence level, to compare the groups. 

## 3. Results

### 3.1. Glucose Diffusion

The Mann-Whitney showed a statistically significant difference among the two groups and the two controls (*P* < 0.001). The negative controls were statistically different from both material groups which were in turn different from the positive controls. The negative controls were below the detection limit of the glucose test and positive-control glucose diffusion was measured as 0.450 ± 0.320 g/L (Table 3). Glucose concentrations of 0.074 ± 0.035 g/L and 0.080 ± 0.032 g/L were recorded for the Biodentine group and the Ionolux group, respectively. The test failed to detect a statistically significant difference between both cements; the two materials thus allowed similar glucose diffusion at the interface between the restorative materials and the dentin walls. 

## 4. Discussion

This study compared the *in vitro* marginal integrity of open-sandwich restorations based on aged calcium silicate-cement or resin-modified glass ionomer and concluded that no statistically significant difference exists between the two treatments. The new calcium silicate-based material performed as well as the resin-modified glass ionomer cement. In addition, the calcium silicate cement did not require specific preparation of the dentin walls.

The good marginal integrity of open-sandwich restorations filled with Biodentine is likely due to the outstanding ability of the calcium silicate materials to form hydroxyapatite crystals at the surface [23]. When formed at the interface between the restorative material and the dentin walls, these crystals may contribute to the sealing efficiency of the material. Just after mixing, the calcium silicate particles of Biodentine, like all calcium silicate materials, react with water to form a high-pH solution containing Ca^2+^, OH^−^, and silicate ions. In the saturated layer, the calcium silicate hydrate gel precipitates on the cement particles, whereas calcium hydroxide nucleates [24]. The calcium silicate hydrate gel polymerizes over time to form a solid network, while the release of calcium hydroxide increases the alkalinity of the surrounding medium. Saliva, like other body fluids, contains phosphate ions [25]; an interaction between the phosphate ions of the storage solution and the calcium silicate-based cements leads to the formation of apatite deposits that may increase the sealing efficiency of the material [23]. A micro-Raman spectroscopy study revealed crystalline apatite and calcite at the surface of a calcium silicate cement stored in phosphate-buffered saline for 28 days [26]. Therefore, the soaking solution needed to be buffered to prevent alkaline erosion of the cement, needed to be frequently renewed to maintain the ongoing diffusion from the restorative material toward the surrounding medium and needed to contain phosphate ions to permit hydroxyapatite formation [27]. Since the effects of various anions and cations on the dissolution process of calcium silicate-based cement have not yet been explored, it was decided to store the teeth in phosphate-buffered saline instead of artificial saliva [28]. In addition to the formation of apatite crystals, the nanostructure of the calcium silicate hydrate may also explain the good sealing qualities of the calcium silicate cement [29]. The small size of forming gels may contribute to better spreading of the material onto the surface and better fitting to dentin walls; a slight expansion of the calcium silicate-based materials in water and phosphate-buffered saline has also been demonstrated [30].

In order to achieve a valid comparison, the experimental protocol must reproduce optimal clinical conditions. Thus, prior to microleakage assessment, in this study the teeth underwent a mechanical loading associated with simultaneous thermocycling that was performed with a device already in use for the assessment of composite resistance to oral wear [31] and restoration microleakage [22]. Previously, simultaneous load cycling and thermocycling were decisive factors in microleakage evaluation [32]. Axial loading was performed with a 50-N force in the present study because class II restorations were evaluated [33]; this loading corresponds to the process that occurs *in vivo* on natural teeth when clenching in centric occlusion [34]. The teeth were stored at 4°C which was not clinically relevant and may have reduced the chemical reaction rate. Nevertheless, a low storage temperature was indispensable because pilot studies had shown that it was difficult to control bacterial growth for periods as long as one year. This is why 0.5% chloramine.T was added to the storage medium. In addition, this was counterbalanced by a very long storage time. Thermomechanocycling was performed before aging when the dentin/material interface was not yet filled with material resulting from calcium silicate or resin-modified glass ionomer cement dissolution. This likely increased the thermomechanocycling efficiency without favoring any group because both materials have a long setting time [35].

Since the dye penetration studies are questionable, a new assessment method was applied to evaluate marginal integrity. The glucose test was first proposed in dentistry to detect apical leakage of endodontic treatments [36]. With this model, it was possible to quantify the microleakage, an improvement over earlier qualitative scoring methods such as dye penetration. Glucose was chosen as a tracer because of its small molecular size (molecular weight 180 g/mol) and because it serves as a nutrient for bacteria. If glucose were able to diffuse from the oral cavity to the pulpal space, bacteria that survive cavity preparation and filling could multiply and cause recurrent caries and pulpal inflammation. The enzymatic method used in the present work is very sensitive. First, glucose is oxidized to gluconic acid and hydrogen peroxide by glucose oxidase. Second, hydrogen peroxide reacts with o-dianisidine in the presence of peroxidase to form a colored product. Third, oxidized o-dianisidine reacts with sulfuric acid to form a more stable colored product. The intensity of the pink color measured at 540 nm is proportional to the original glucose concentration. The detection limit of the method was 2 × 10^−3^ g/L which is very low compared to the data recorded in the present study and warrants the quality of controls. The difference of levels of magnitude of the data recorded with the negative and positive controls showed that the method is valid for the assessment of open-sandwich restoration marginal integrity.

It can be concluded from the present study that resin-modified glass ionomer cement and the calcium silicate cement allowed similar glucose diffusion at the interface between the restorative materials and the dentin walls. The calcium silicate cements may become a material of choice for restorative dentistry in the coming years and compete with the resin-modified glass ionomer cement.

## Figures and Tables

**Table 1:** Chemical composition of Biodentine, Septodont, St Maure des Fossés, France.

(i) Tricalcium silicate (CaO)_3_ SiO_2 _	
(ii) Calcium carbonate: CaCO_3_	
(iii) Zirconium dioxide: ZrO_2_	

Liquid:	

(i) Calcium chloride: CaCl_2_	
(ii) Water	
(iii) Water reducing agent	

**Table 2:** Composition (in g/L) of the Dulbecco's phosphate buffered saline used for the study.

(i) CaCl_2_·2H_2_O:	0.133
(ii) MgCl_2_·6H2O:	0.1
(iii) KCl:	0.2
(iv) KH_2_PO_4_:	0.2
(v) NaCl:	8.0
(vi) Na_2_HPO_4_:	1.15

**Table 3:** Glucose concentration in the receiving medium after 1 h diffusion through the gingival margin of open-sandwich restorations filled with a calcium silicate-cement or a resin-modified glass ionomer cement. A statistically significant difference was found among the four groups. The groups with the same superscript letter were not statistically different.

Material	Glucose concentration (g/L)
Calcium silicate cement	0.074 ± 0.035^b^
Resin-modified glass ionomer cement	0.080 ± 0.032^b^
Positive control	0.450 ± 0.320^c^
Negative control	0 ± 0.002^a^
